# The effect of peer support on psychological rehabilitation in injured collegiate athletes: the mediating roles of resilience and perceived stress

**DOI:** 10.3389/fpsyg.2025.1567812

**Published:** 2025-10-02

**Authors:** Jiawei Liu, Jianghong Zhao, Zhenhao Wang

**Affiliations:** ^1^School of Physical Education, Shanghai University of Sport, Shanghai, China; ^2^Center of Physical Education, Xi'an Jiaotong University, Xi’an, Shaanxi, China; ^3^Faculty of Humanities and Social Sciences, Xi'an Jiaotong University, Xi’an, Shaanxi, China

**Keywords:** peer support, sports injury rehabilitation, collegiate athletes, psychological resilience, perceived stress, parallel mediation

## Abstract

**Background:**

Sports injuries are a significant concern for collegiate athletes, often leading to mental issues, including anxiety, depression, and even somatization, due to the combined impact of physical limitations and academic pressure. Peer support interventions (PSI) provide an innovative approach by leveraging shared experiences, emotional support, and coping strategies to facilitate psychological rehabilitation. However, empirical evidence on their efficacy in injured collegiate athletes remains limited. This study aims to assess the efficacy of PSI in facilitating psychological rehabilitation among injured collegiate athletes.

**Method:**

A randomized controlled trial was conducted with 51 injured collegiate athletes assigned to either experiment group (PSI, *n* = 25) or control group (CON, *n* = 26). The PSI group participated in a 6-week peer support program, while the control group received low-intensity mental health education. Psychological resilience, perceived stress, and mood states were assessed at baseline (T1), 3 weeks (T2), and 6 weeks (T3). Additionally, non-standardized qualitative interviews (*n* = 10) explored participants’ subjective experiences and the mechanisms underlying PSI effectiveness.

**Results:**

Both groups showed varying degrees of improvement across all measured indicators compared to baseline, while the PSI group demonstrated higher effect sizes. Compared to the control group, the PSI significantly enhanced psychological resilience (*η^2^_p_* = 0.349) and reduced perceived stress (*η^2^_p_* = 0.572), thereby improving positive moods (e.g., activity and calmness) and reducing negative moods (e.g., anger and depression). Path analysis further revealed that psychological resilience and perceived stress functioned as two parallel mediators through which peer support facilitated improvements in mental health outcomes (*β_N_* = −0.864, *β_P_* = 0.912, *p* < 0.001).

**Conclusion:**

This study validates PSI as a cost-effective and highly interactive psychological intervention that enhances psychological resilience and alleviates stress in injured collegiate athletes, contributing to overall mental wellbeing. Further larger trials are required to confirm these findings.

**Clinical trial registration:**

https://www.chictr.org.cn/showproj.html?proj=251796, Identifier XJTU1AF2024LSYY-224

## 1 Introduction

Sports injuries are a prevalent issue among athletes (Putukian, 2016). Empirical research indicates that such injuries not only affect athletes’ competitive performance but may also cause physiological limitations, triggering mental health issues such as anxiety, depression, and frustration (Wolanin et al., 2016). In severe cases, they can even lead to conditions like substance abuse and subclinical eating disorders (Lederer and Hoban, 2022). The American Medical Society for Sports Medicine explicitly states that “athletes recovering from major illnesses or injuries should be screened for depression and anxiety, followed by psychological interventions to facilitate their recovery” (Chang et al., 2020). Collegiate athletes, who must simultaneously manage both competitive pressures and academic demands, exhibit greater complexity and uniqueness in emotional functioning, stress management, and psychological adaptation compared to non-student athletes (Lam et al., 2013). Consequently, they are at a higher risk of depression and anxiety symptoms, with research indicates that approximately 20% of collegiate athletes experience severe mental health issues following sports injuries (Li et al., 2017; Yang et al., 2007). If these psychological challenges are not effectively addressed in a timely manner, they may not only delay physical recovery but also increase the risk of reinjury due to heightened neural sensitivity, ultimately exerting long-term negative effects on both overall health and athletic performance (Brand et al., 2013).

To support the psychological rehabilitation of collegiate athletes, defined as the structured restoration of psychological functioning and mental well-being (Palmer and Wegener, 2003), extensive research and practical exploration have led experts to develop various intervention strategies, including individual psychotherapy based on cognitive-behavioral therapy (CBT), breathing relaxation techniques, and mindfulness training, all of which have demonstrated significant efficacy. For instance, psychological counseling rooted in CBT enables athletes to more accurately identify and regulate their psychological states (Sutcliffe and Greenberger, 2020), while breathing relaxation and mindfulness interventions enhance self-regulation abilities and reduce anxiety levels by modulating physiological and psychological responses (Gross et al., 2018; Hanton et al., 2015).

In intervention studies such as cognitive-behavioral therapy, mindfulness training, identifying and strengthening key psychological factors, such as mindfulness levels, perceived stress, psychological resilience, and subjective well-being, is central to psychological rehabilitation (Kegelaers et al., 2024). Among these factors, perceived stress and psychological resilience are particularly important, as both have been shown to influence mental adjustment and rehabilitation outcomes (Lehrer et al., 2020). Perceived stress, defined as an individual’s subjective appraisal of external stressors, not only positively predicts mental health issues such as anxiety and depression but also negatively influences mental well-being and life satisfaction (Li and Ma, 2019). Psychological resilience, meanwhile, serves as a crucial protective factor in mood regulation, reflecting an individual’s ability to “bounce back” when confronted with significant stress and adversity (Southwick et al., 2014). Notably, resilience is not a static trait but rather a dynamic process that can be cultivated and strengthened through targeted interventions (Steen et al., 2014). In this study, perceived stress and psychological resilience are regarded as central indicators of psychological rehabilitation, functioning as key mediators through which peer support contributes to improved adjustment and reduced risk of future injuries.

Currently, Chinese universities predominantly rely on psychological interventions led by counseling teachers to address psychological issues among collegiate athletes (Hong, 2018). These interventions often take the form of one-on-one psychological counseling or mental health lectures. While these interventions help alleviate individual psychological issues and promote mental recovery to some extent, they still have several limitations. First, this symptom-focused approach primarily emphasizes problem-solving while overlooking the cultivation of students’ intrinsic psychological resilience and strengths, making it difficult to achieve long-term improvements in mental health. Second, these interventions are typically implemented only after psychological issues have become evident or severe, with mechanisms for early prevention and proactive detection still underdeveloped. This has led to an increasing disconnect between the interventions and the actual needs of the students (Liu et al., 2022). Finally, the professional-led model may create a power imbalance, resulting in a lack of relatability and trust between students and counselors, which can undermine the effectiveness of the interventions. Additionally, the high costs and limited accessibility of such interventions have restricted their widespread application in practice (Sun et al., 2023).

Research indicates that injured collegiate athletes often rely on personal coping strategies rather than proactively seeking mental health support due to barriers such as stigma, insufficient mental health literacy, and negative past experiences with seeking help (Gulliver et al., 2012; Moreland et al., 2018). Moreover, most collegiate athletes have not yet developed mature psychological coping mechanisms and often perceive help-seeking as a sign of “weakness” or “lack of resilience,” believing they can “overcome” psychological barriers in the same way they confront physical challenges (Putukian, 2016). This deeply ingrained mindset further hinders the effective implementation of psychological interventions.

Peer support is a “quasi-counseling” model centered on peer interaction, in which selected and briefly trained individuals provide psychological support, comfort, and guidance to peers in need of mental health assistance (Dennis, 2003). Grounded in social support theory, peer support emphasizes the establishment of supportive relationships among individuals with similar backgrounds and experiences. Through emotional support (direct effect), mitigating the impact of stressors (buffering effect), facilitating the exchange of health-related and self-management information, and offering positive role modeling (mediating effect). By fostering emotional resonance among peers, this approach helps alleviate psychological stress, restore a sense of belonging, and rebuild confidence (Watson, 2019). Due to its inherent high relatability, proactive intervention timing, and superior cost-effectiveness, peer support has been widely applied in various contexts. Existing research demonstrates that peer interventions targeting groups such as individuals with substance use disorders (O’Connell et al., 2020), early-stage psychosis patients (Galloway and Pistrang, 2019), college students (Byrom, 2018), and military veterans (Weir et al., 2019) have shown significant effectiveness in promoting mental health, shortening recovery times and achieving comprehensive recovery outcomes.

Given the prevalent limitations of traditional psychological interventions, such as a lack of proactivity, poor timeliness, and insufficient demand specificity, this study innovatively applies the peer support framework—characterized by strong relatability, timely responsiveness, and clear demand orientation—to the psychological rehabilitation of collegiate athletes following sports injuries (Lloyd-Evans et al., 2014). The study selects two key psychological factors, psychological resilience and perceived stress, which significantly influence individual mood states, as primary mediating indicators in the promotion of psychological rehabilitation through peer support. Specifically, the research aims to achieve the following objectives: (1) To examine the effectiveness of peer support in promoting psychological rehabilitation among injured collegiate athletes; (2) To elucidate the mechanisms through which peer support influences key psychological factors in the rehabilitation process; (3) To reveal the parallel mediation effect of psychological resilience and perceived stress in the relationship between peer support and psychological rehabilitation in injured collegiate athletes.

## 2 Research participants and methods

### 2.1 Subsection

Sample Size Estimation: The minimum required sample size was calculated using G*Power 3.1.9.2. Based on prior studies (Bryan and Arkowitz, 2015), the interaction effect size was set to a moderate level (*f* = 0.25), with time (baseline, mid-test, post-test) as a within-subject factor, an alpha level of 0.05, and statistical power (1-*β*) = 0.90. The calculation determined that a total of at least 36 participants were required, divided between the peer support intervention group (PSIG) and the control group (CON).

**Figure 1:**
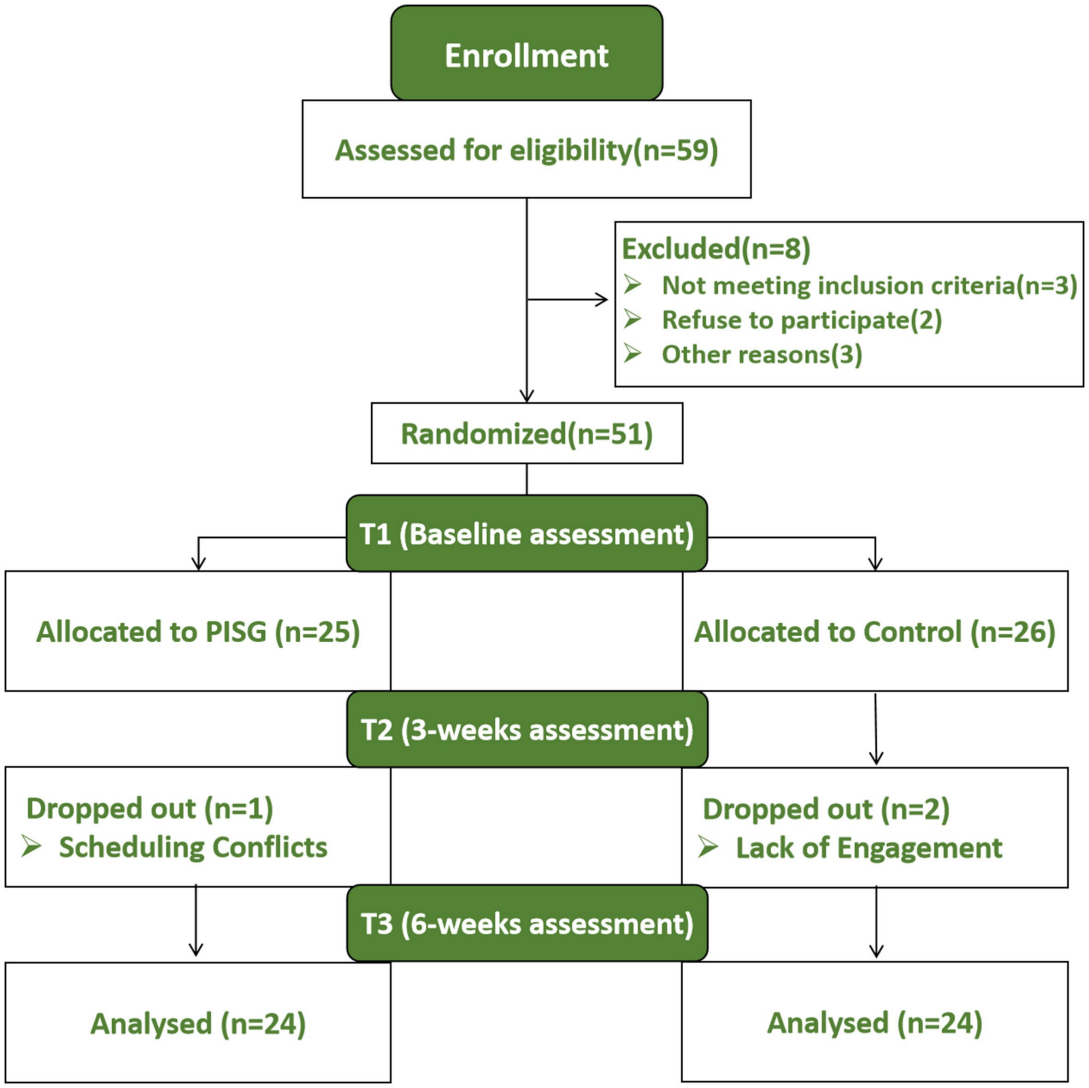
Consort flow diagram.

Participants were recruited through advertisements distributed via WeChat public accounts, email, and internal recommendations from sports teams. Prior to enrollment, participants completed the Self-Rating Anxiety Scale (SAS) and reported any history of major illnesses or chronic diseases, including psychiatric disorders. The inclusion criteria were as follows: (1) No self-reported history of major illnesses or chronic diseases (e.g., infectious diseases, heart disease, cancer, or psychiatric disorders); (2) Currently enrolled collegiate student-athletes; (3) Having sustained a sports-related injury and being in the rehabilitation (non-acute) phase; (4) Self-reported mild emotional symptoms and elevated stress (SAS < 60, subclinical levels). Exclusion criteria included: (1) Self-reported history of clinically diagnosed psychiatric or psychological disorders (e.g., major depressive disorder, generalized anxiety disorder); (2) Being in the acute phase of an injury, to avoid confounding due to unstable physical and clinical conditions; (3) Not meeting the criteria for enrolled collegiate students or being over 25 years old (Figure 1).

A total of 51 collegiate athletes from various universities in Shaanxi were recruited as research participants. The participants were randomly assigned to the experimental group (PSIG, *n* = 25) and the control group (CON, *n* = 26). Three participants subsequently dropped out of the study. During screening, participants reported common sports-related injuries such as ligament sprains or strains, muscle injuries, and meniscal or tendon damage. Before testing, all participants were informed about the research content by their counselors and provided written informed consent.

### 2.2 Research instruments

#### 2.2.1 Connor-Davidson resilience scale (CD-RISC)

Connor and Davidson (2003) developed the Connor-Davidson Resilience Scale (CD-RISC) to assess individual levels of psychological resilience. Yu and Zhang (2007) later adapted this scale into Chinese version. The scale primarily consists of three dimensions: tenacity, strength, and optimism. Example items include: “I am able to adapt when changes occur” and “I have become stronger through difficult experiences.” Responses are rated on a 5-point Likert scale (1 = “Not confident at all” to 5 = “Very confident”). The total score is calculated as the mean score of all 25 items, with higher scores indicating a higher level of trait psychological resilience. In this study, the internal consistency was 0.87 for the total scale, and 0.80, 0.66, and 0.63 for the subscales of Tenacity, Strength, and Optimism, respectively.

#### 2.2.2 Mood survey scale (BFS)

This study utilized the BFS Mood Survey Scale (Befindlichkeitsskalen), a German-language tool for measuring and describing mental states, was used to better reflect the relationship between mental state and subjects’ moods. Developed by Abele and Brehm (1986), the BFS scale is widely used in research within German-speaking countries. The BFS scale consists of eight subscales, categorized into positive subscales (activity, pleasure, calmness, and thoughtfulness) and negative subscales (anger, excitement, depression, and lack of vitality). Each subscale includes five items, resulting in a total of 40 items. The scale was translated into Chinese by Si and Huang (1997), and its reliability and validity were tested, demonstrating good theoretical and construct validity. In this study, the internal consistency coefficients for the eight subscales ranged from 0.65 to 0.86, indicating its suitability for assessing mood states in the psychological rehabilitation of injured collegiate athletes.

#### 2.2.3 Chinese perceived stress scale (CPSS)

The Perceived Stress Scale (PSS), originally developed by Cohen et al. (1983), was adapted into Chinese by Yang and Huang (2003). It is designed to assess an individual’s subjective perception of stress, identifying health-risk psychological stress (HRS), and is currently the most widely used scale in China. The CPSS consists of 14 items (including 7 reverse-scored items) and includes two factors: tension and loss of control. Responses are rated on a 5-point scale (0 = “Never” to 4 = “Always”), with a total score range of 0 to 56, where higher scores indicate greater perceived stress levels. In the present study, the internal consistency was 0.75 for the total scale, with alpha values of 0.62 and 0.61 for the Tension and Loss of Control subscales, respectively.

### 2.3 Research design

In terms of experimental design, this study employed a 3 (baseline, mid-test, post-test) × 2 (Experimental group, Control group) single-blind, two-group, equal design. The experimental group received peer support (PS) interventions, while the control group received low-intensity psychological interventions. The intervention protocol lasted approximately 6 weeks (45 days), comprising 8 sessions delivered 1–2 times per week, each lasting 80 to 120 min. Questionnaire surveys were administered at three time points: baseline (T1), mid-test (T2), and post-test (T3). The experimental group completed paper-based questionnaires in person, while the control group completed the same questionnaires online via WeChat, with both formats designed to ensure equivalent item presentation and anonymous responses to minimize mode-related bias (Podsakoff et al., 2003). By comparing the psychological state trends under intervention conditions in both groups, this study scientifically assessed the impact of peer support in promoting psychological rehabilitation among students after sports injuries. To gain a more comprehensive understanding of participants’ subjective experiences and behavioral patterns, a small-scale, non-standardized interview of the experimental group (*n* = 10) was conducted upon completion of the intervention. The interviews were facilitated by two researchers, with detailed field notes recorded during the process.

This study was approved by the Ethics Committee of the First Affiliated Hospital of Xi’an Jiaotong University (Approval Number: XJTU1AF2024LSYY-224) and was conducted in accordance with the principles of the Helsinki Declaration. All participants provided written informed consent prior to their involvement in the study.

#### 2.3.1 Peer counsellor

Before initiating peer support interventions, the establishment of a scientifically rigorous selection and training process is a critical prerequisite for ensuring their effectiveness. In this study, one peer counselor (male, age 22), a collegiate basketball player with a history of ligament injury, was selected through a rigorous screening process emphasizing prior experience with sports injury rehabilitation. This selection strategy was intended to maximize empathic attunement and contextual understanding of the target population’s psychological needs. Drawing on shared lived experiences, the peer counselor was well-positioned to build a trust-based therapeutic rapport with injured athletes, providing a solid emotional foundation for psychological assistance.

Prior to beginning their roles, peer counselors underwent systematic, phased training aimed at integrating theoretical foundations with practical skills in supportive relationships and peer assistance (Becker and Zarit, 1978; Brammer, 1973). The training lasted for 4 weeks and emphasized four key modules: (1) active listening, prioritizing attentive listening over speaking; (2) fostering strong interpersonal connections; (3) demonstrating empathy and providing emotional support; (4) encouraging injured individuals to reengage in physical activity, such as reframing stressful relationships or planning activities to mitigate social isolation. The curriculum covered essential psychological concepts (e.g., emotional regulation mechanisms and cognitive-behavioral theory), practical communication techniques (e.g., nonjudgmental listening, emotional recognition, and constructive feedback), and basic crisis intervention protocols (e.g., standardized responses to acute psychological issues). Guided by Bordin’s supervision process model (Bordin, 1983), the training was conducted under the supervision of experienced psychological educators, who provided ongoing feedback and support to enhance the counselors’ professional competence and problem-solving skills. By rigorously selecting and training peer counselors, the study ensured the intervention’s professionalism and consistency while offering empirical evidence to advance and expand the application of the peer support model.

#### 2.3.2 Intervention program for the PSI group

**Figure 2:**
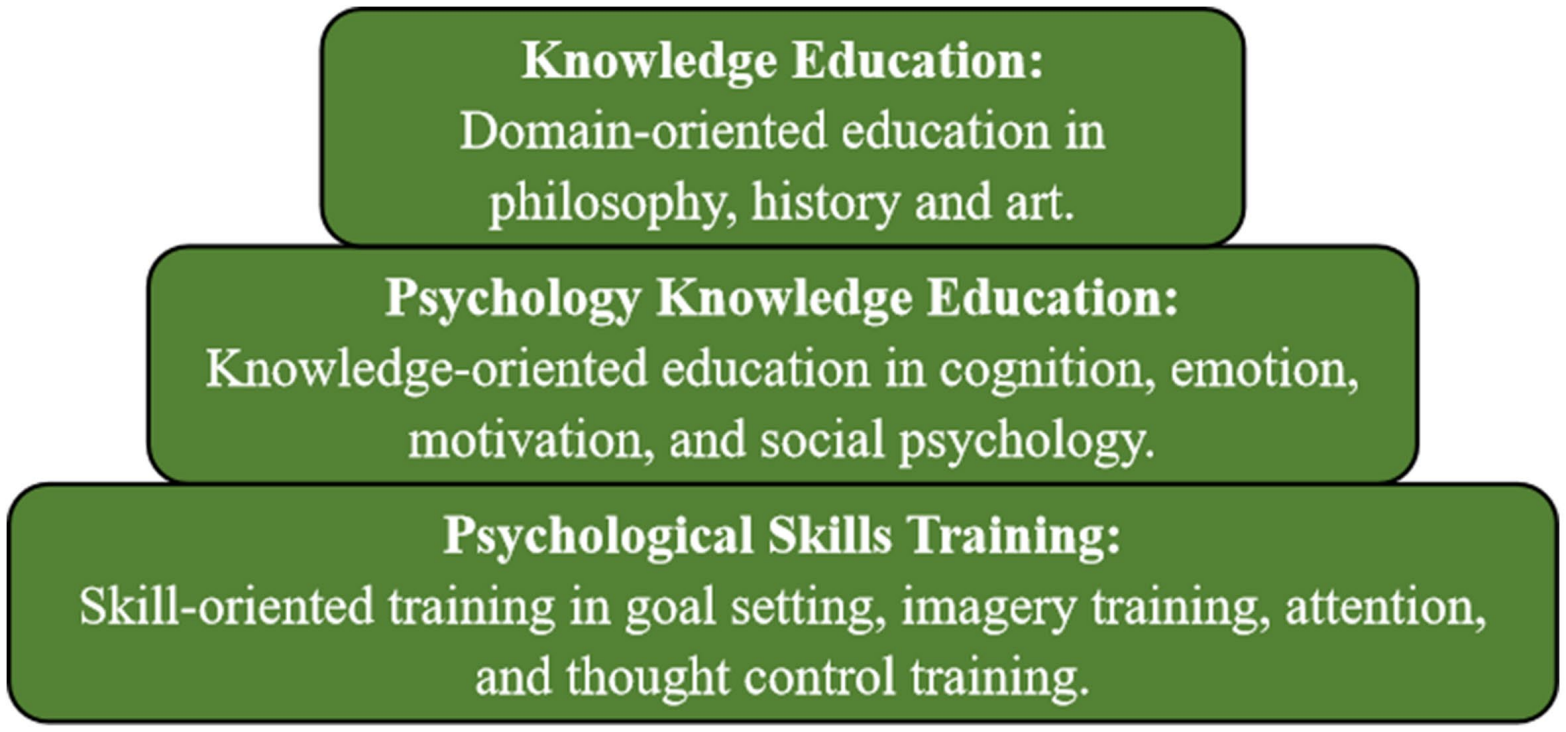
Chinese athletes’ psychological construction system.

Members of the experimental group will undergo peer psychological intervention, which integrates Western skill training theories and techniques with traditional Chinese cultural and philosophical elements. Drawing from relevant literature, structured interviews, and past clinical experience, the intervention design employs the Chinese Athlete Psychological Construction System (Zhang and Zhang, 2011) as its theoretical foundation (Figure 2). The intervention includes activities such as interactive games, group discussions, role-playing, and behavioral exercises. Peer counselors lead the program, fostering deep connections among participants, promoting effective coping with psychological distress, and gradually enhancing psychological resilience through a structured sequence of activities. The specific activity plan is outlined below (Table 1):

**Table 1:** Peer support intervention programs.

Phase	Session	Theme	Rationale
Initiation	1	Icebreaker activities	Facilitates interaction and trust among participants, fostering group cohesion. Reduces feelings of unfamiliarity, improve communication skills
	2	Mental health education	Increases awareness of mental health issues, strengthening self-regulation abilities. Provides scientifically grounded knowledge on maintaining mental health
Familiarization	3	Group discuss	Encourages self-expression and reflection, promoting mental recovery.
	4	Seminar on sports injury management	Educates participants on injury prevention and recovery, reducing psychological stress related to injury fears.
Learning	5	Emotions and coping	Strengthens coping mechanisms essential for psychological resilience and recovery.
	6	Skill acquisition	Provides practical tools like mindfulness to improve emotional control and mental stability
Summarization	7	Recreational games	Offers stress relief and relaxation, fostering a positive mood state conducive to recovery. Strengthens social bonds and resilience through group interaction.
	8	Outlook	Encourages goal-setting, motivating participants to focus on their psychological and physical recovery.

##### 2.3.2.1 Phase 1: icebreaking activities–facilitating interpersonal connection and environmental adaptation

As the initial step of the intervention series, icebreaking activities aim to reduce unfamiliarity among participants and foster mutual understanding. Carefully structured interactive tasks, such as guided self-introductions, group discussions on shared interests, and non-competitive activities like “Two Truths and a Lie,” create an open and supportive atmosphere. In structured small-group discussions, participants collaboratively establish group norms and expectations for the peer intervention program, ensuring a safe and inclusive environment. By fostering early group cohesion and psychological safety, this phase lays a strong foundation for meaningful interactions and mutual support in subsequent sessions.

##### 2.3.2.2 Phase 2: deepening awareness–addressing psychological trauma and sharing coping strategies

This phase focuses on encouraging participants to explore and articulate their psychological experiences related to sports injuries in a structured and supportive manner. Sessions begin with guided self-reflection exercises, where participants document their thoughts before engaging in group discussions. Facilitated discussions follow predefined thematic prompts (e.g., injury-related fears, setbacks, and coping mechanisms) to ensure a systematic exploration of psychological trauma. Confidential storytelling and structured peer-sharing sessions, conducted in accordance with established psychological safety protocols, help participants process emotional challenges and recognize common struggles. Additionally, participants engage in coping strategy workshops where they exchange personal experiences and explore practical techniques for stress management, emotional regulation, and injury resilience. By integrating personal narratives with proactive knowledge acquisition, this phase empowers participants to develop a stronger awareness of injury prevention and mental recovery strategies.

##### 2.3.2.3 Phase 3: skill acquisition–practical psychological adjustment techniques

This phase introduces empirically validated psychological intervention techniques, ensuring participants acquire structured and applicable psychological skills. Workshops follow a step-by-step framework, beginning with an instructional component where facilitators introduce core psychological techniques, such as Cognitive Behavioral Therapy (CBT) and Acceptance and Commitment Therapy (ACT). Participants then engage in guided exercises, including “Cognitive Restructuring,” “Positive Self-Affirmation,” “Mindful Acceptance,” among others. By mastering and internalizing these psychological skills, participants can integrate them effectively into daily life, improving emotional regulation, resilience, and confidence in physical rehabilitation.

##### 2.3.2.4 Phase 4: consolidating outcomes–collaboration, communication, and stress alleviation

In the final phase, interactive team-building activities and psychological exercises are used to reinforce group cohesion and provide a structured opportunity for stress relief. Engaging cooperative challenges encourage participants to apply their acquired skills in a dynamic, supportive setting, while reflective discussions help them consolidate their progress. To facilitate long-term recovery planning, participants engage in a structured goal-setting process using the SMART (Specific, Measurable, Achievable, Relevant, Time-bound) framework. They define personalized recovery objectives that are concrete, realistic, and time-bound, ensuring clear benchmarks for progress. These goals are visually represented through written or graphical formats, enhancing motivation and accountability. By fostering a positive and forward-looking mindset, this phase ensures that participants transition from the intervention with strengthened psychological resilience and a clear pathway for sustained recovery.

#### 2.3.3 Intervention program for the control group

In accordance with ethical research principles for working with vulnerable populations (American Psychological Association, 2017), participants in the control group received a low-intensity psychological intervention to ensure basic psychological support during rehabilitation. This included weekly email updates with general motivational content and stress management strategies, as well as brief online group sessions focused on basic psychological health and emotional regulation.

### 2.4 Statistical methods

A one-way analysis of variance (ANOVA) was conducted to compare pre-test measures between groups. For variables showing significant differences, a covariance analysis was performed, using baseline values as covariates to assess group differences in post-test measurements. A two-way (Group × Time) repeated measures ANOVA was used to examine the interaction and main effects of group and time. The *F*-test was applied to assess significant differences between the expected values of the two groups, and partial η^2^ was calculated to determine the effect size of the ANOVA, categorized as small (0.04), medium (0.25), and large (0.64) (Ferguson, 2016). Data are presented as Mean ± SD unless otherwise specified, with statistical significance set at *p* < 0.05. All statistical analyses were performed using IBM SPSS version 29 (IBM, Armonk, NY, USA).

## 3 Analysis of research results

### 3.1 Control and testing for common method bias

To control for procedural bias, the order of the questionnaire items was counterbalanced, and participants were informed in advance about the purpose, content, significance, anonymity, and academic use of the survey. Therefore, Harman’s single-factor test was conducted to examine common method bias. The results showed that the first factor accounted for 24.82% of the total variance, indicating that common method bias was not a significant concern in the present study. The Shapiro–Wilk test was used to assess the normality of all variables at T1, T2, and T3. The results showed that kurtosis values were below 10 and skewness values were below 3, indicating that the data met the assumptions of normality and justifying the use of parametric tests.

### 3.2 Baseline characteristics

Homogeneity testing was conducted using an *t*-test for variance, as shown in Table 2. Except for a subscale of the BFS (Excitement*, t* = 2.338, *p* = 0.024), no statistically significant differences were found in age, gender, perceived stress, psychological resilience, or mood state scores across the two groups (*p* > 0.05). This suggests that, following random assignment, there were no significant differences in demographic or clinical characteristics between the groups. Both groups met the path standards for randomized controlled trials and were comparable in terms of clinical characteristics.

**Table 2:** Baseline outcome measures at time point (T1) (*n* = 51).

Variable	CON (n = 26)	PSIG (n = 25)	*t-test*
	$\bar{X}$ *(SD)*	$\bar{X}$ *(SD)*	
Age (years old)	20.85 ± 1.80	21.04 ± 1.84	−0.38
Sex, *n* (%)			
Male	15 (57.7)	14 (56.0)	
Female	11 (42.3)	11 (44.0)	
CD-RISC			
Te	1.39 ± 0.20	1.36 ± 0.14	0.573
St	0.86 ± 0.13	0.87 ± 0.10	−0.405
Op	0.41 ± 0.06	0.40 ± 0.06	0.658
Total	2.65 ± 0.33	2.63 ± 0.26	0.303
CPSS			
Tn	21.69 ± 1.67	21.76 ± 2.42	−0.117
LC	21.46 ± 2.30	21.24 ± 2.40	0.336
Total	43.15 ± 3.08	43.00 ± 4.09	0.152
BFS			
Ac	9.81 ± 2.02	9.20 ± 1.29	1.285
Pl	11.08 ± 1.72	10.28 ± 1.28	1.885
Ca	10.04 ± 2.44	9.68 ± 1.49	0.635
Th	10.27 ± 1.43	10.60 ± 2.06	−0.663
An	19.77 ± 2.32	18.76 ± 1.45	1.87
Ex	20.19 ± 1.55	19.28 ± 1.21	2.338^*^
De	19.23 ± 1.73	18.40 ± 2.33	1.443
LV	20.65 ± 2.06	19.68 ± 1.70	1.838

* < 0.05; ** < 0.01; *** < 0.001; PSIG, Peer Support Intervention Group; Te = Tenacity; St = Strength; Op = Optimism; Tn = Tension; Lc = Loss of Control; Ac = Activity; Pl = Pleasure; Ca = Calmness; Th = Thoughtfulness; An = Anger; Ex = Excitement; De = Depression; LV = Lack of Vitality.

### 3.3 Repeated measures ANOVA

Repeated measures analysis of variance (ANOVA) was conducted to examine the scores for perceived stress, mood state, and psychological resilience, using a 2 (group: experimental group, control group) × 3 (time: baseline, mid-test, post-test) design (Table 3).

**Table 3:** Estimated marginal means and the group effect at time point 2 (T2) and time point 3 (T3) from ANCOVA.

Variable	T1	T2 (3 weeks)				T3 (6 weeks)			
	Adjusted baseline	CON (*n* = 24)	PSIG (*n* = 24)	*F-test*	*η^2^_p_*	CON (n = 24)	PSIG (n = 24)	*F-test*	*η^2^_p_*
	*M*	*M ± SD*	*M ± SD*			*M ± SD*	*M ± SD*		
CD-RISC									
Te	1.38	1.40 ± 0.10	1.52 ± 0.12	14.12^***^	0.23	1.48 ± 0.14	1.66 ± 0.11	25.2^***^	0.35
St	0.86	0.89 ± 0.10	0.93 ± 0.07	2.37	0.05	0.90 ± 0.09	0.97 ± 0.03	12.91^***^	0.22
Op	0.40	0.40 ± 0.05	0.44 ± 0.07	4.576^*^	0.09	0.44 ± 0.06	0.50 ± 0.06	11.25^**^	0.20
Total	2.64	2.69 ± 0.20	2.90 ± 0.20	11.96^**^	0.21	2.82 ± 0.25	3.13 ± 0.17	24.66^***^	0.35
CPSS									
Tn	21.73	17.96 ± 1.68	17.50 ± 1.91	0.778	0.02	17.25 ± 2.01	14.42 ± 2.28	20.87^***^	0.44
LC	21.35	19.92 ± 1.89	17.96 ± 2.31	10.34^**^	0.18	18.13 ± 1.65	15.17 ± 1.79	35.53^***^	0.31
Total	43.12	37.88 ± 2.11	35.46 ± 2.65	12.18^**^	0.21	35.38 ± 2.39	29.58 ± 2.72	61.42^***^	0.57
BFS									
Ac	9.51	11.75 ± 1.75	12.88 ± 1.39	6.07^*^	0.12	12.96 ± 3.17	15.79 ± 1.61	15.23^***^	0.25
Pl	10.69	12.33 ± 1.63	13.25 ± 1.19	4.94^*^	0.10	13.42 ± 1.98	15.38 ± 1.91	12.20^**^	0.21
Ca	9.86	11.13 ± 2.11	12.04 ± 1.30	3.28	0.07	13.08 ± 2.28	14.96 ± 1.90	9.57^**^	0.17
Th	10.43	12.08 ± 1.84	13.54 ± 1.35	9.80^**^	0.18	13.29 ± 1.92	15.25 ± 1.70	13.98^***^	0.23
An	19.27	17.42 ± 2.32	16.04 ± 1.63	5.65^*^	0.11	16.79 ± 2.47	14.00 ± 2.83	13.28^***^	0.22
Ex	19.75	18.29 ± 2.01	17.04 ± 1.20	6.85^*^	0.13	17.21 ± 2.13	14.21 ± 2.17	23.44^***^	0.34
De	18.82	17.42 ± 1.79	16.17 ± 1.34	7.49^**^	0.14	17.04 ± 2.33	13.58 ± 1.91	31.62^***^	0.41
LV	20.18	18.88 ± 2.44	17.75 ± 1.19	4.13^*^	0.08	17.54 ± 1.86	15.00 ± 1.25	30.75^***^	0.40

* < 0.05; ** < 0.01; *** < 0.001; PSIG, Peer Support Intervention Group; Te = Tenacity; St = Strength; Op = Optimism; Tn = Tension; Lc = Loss of Control; Ac = Activity; Pl = Pleasure; Ca = Calmness; Th = Thoughtfulness; An = Anger; Ex = Excitement; De = Depression; LV = Lack of Vitality.

The main effect of group (experimental vs. control) on psychological resilience scores was significant (*F* = 11.865, *p* < 0.01, *η^2^_p_* = 0.205), as was the main effect of time (*F* = 32.853, *p* < 0.001, *η^2^_p_* = 0.417). Additionally, the interaction effect between group and time was significant (*F* = 6.855, *p* < 0.01, *η^2^_p_* = 0.13). A within-group analysis revealed a significant difference in resilience scores from T1 to T3 in the experimental group (*F* = 32.169, *p* < 0.001, *η^2^_p_* = 0.483), while no significant change was observed in the control group over the same period (*p* > 0.05). At baseline (T1), there was no significant difference in scores between the experimental and control groups (*p* > 0.05). However, at T2 and T3, the scores of the two groups differed significantly (*F* = 11.96, *p* < 0.01; *F* = 24.66, *p* < 0.001), with the effect size between the groups progressively increasing (*η^2^_p_* = 0.206 to 0.349). This suggests that peer intervention significantly enhances the psychological resilience of participants, with the intervention’s effect strengthening over time.

The main effects of group, time, and their interaction on perceived stress scores were all significant (*F* = 27.238, *F* = 171.43, *F* = 12.361, *p* < 0.001). In the experimental group, perceived stress scores at T3 were significantly lower than those at the T1 pre-test (*F* = 101.57, *p* < 0.001), indicating a reduction in stress levels following the intervention. Similarly, the control group also showed a significant reduction over this period (*F* = 55.426, *p* < 0.001), with both groups demonstrating lower post-test scores compared to pre-test scores. At both T2 and T3, significant differences between the experimental and control groups were observed (*F* = 12.185, *F* = 61.421, *p* < 0.01), with the effect size between the groups gradually increasing (*η^2^_p_* = 0.209 to 0.572). This indicates that both interventions were effective in reducing perceived stress at different time points, but the experimental group exhibited a more significant improvement compared to the control group.

The main effects of group, time, and their interaction on the negative and positive mood scale scores were all significant (*p* < 0.001). A within-group analysis revealed significant differences in scores from T1 to T3 in both the experimental and control groups (*p* < 0.001), with the experimental group showing significantly higher effect sizes compared to the control group. Between-group comparisons at T2 revealed significant differences on all subscales except calmness (*F* = 3.28, *p* > 0.05), with effect sizes remaining relatively small (*F* = 4.13 ~ 9.80, *p* < 0.05). At T3, the differences between the two groups’ scores progressively increased, with the experimental group demonstrating significant improvement in mood after peer support interventions. All subscales of the Mood Survey Scale showed significant statistical differences (*F* = 9.57 ~ 31.62, *p* < 0.01) and larger effect sizes (*η^2^_p_* = 0.172 ~ 0.407) compared to the control group. This provides strong evidence that peer support significantly improves mood states in collegiate athletes following sports injuries. In contrast, the control group displayed a stagnation phase from T2 to T3, where, except for the pleasure, calmness, thoughtfulness, and lack of vitality indicators, there were no significant differences in the other indicators (*p* > 0.05), and the effect sizes in these indicators showed a diminishing trend (Table 4).

**Table 4:** Two-way repeated measures ANOVA results for the impact of outcome variables.

Dependent variable	SOV	SST	MS	*F*	*η^2^_p_*
RES	Group	1.061	1.061	11.865^**^	0.205
	Times	2.723	1.362	32.853^***^	0.417
	Times × Group	0.568	0.284	6.855^**^	0.130
PSR	Group	277.778	277.778	27.238^***^	0.372
	Times	2704.597	1352.299	171.43^***^	0.788
	Times × Group	195.014	97.507	12.361^***^	0.212
PM	Group	532.84	532.84	23.918^***^	0.342
	Times	6702.875	3351.437	192.765^***^	0.807
	Times × Group	620.931	310.465	17.857^***^	0.280
NM	Group	1620.062	1620.062	71.846^***^	0.610
	Times	5399.681	2699.84	192.867^***^	0.807
	Times × Group	481.792	240.896	17.209^***^	0.272

* < 0.05, ** < 0.01; *** < 0.001; PSI, Peer Support Intervention; RES, Resilience; PSR, Perceived Stress; NM, Negative Mood; PM, Positive Mood.

### 3.4 Correlation analysis

Based on the T3 data, Spearman correlation analysis revealed significant relationships among the variables. Peer support was positively correlated with psychological resilience (*r* = 0.601, *p* < 0.001) and positive mood states (*r* = 0.624, *p* < 0.001), while showing a significant negative correlation with negative mood (*r* = −0.764, *p* < 0.001) and perceived stress (*r* = −0.767, *p* < 0.001). Additionally, psychological resilience was negatively correlated with negative mood (*r* = −0.750, *p* < 0.001) and perceived stress (*r* = −0.645, *p* < 0.001), but positively correlated with positive mood states (*r* = 0.717, *p* < 0.001). These findings indicate significant associations among the variables, making them suitable for further investigation (Table 5).

**Table 5:** Correlation analysis of study variables.

Variable	*M ± SD*	PSI	RES	PSR	PM	NM
PSI		1				
RES	2.973 ± 0.264	0.601^***^	1			
PSR	32.479 ± 3.87	−0.767^***^	−0.645^***^	1		
PM	57.063 ± 7.206	0.624^***^	0.717^***^	−0.739^***^	1	
NM	62.688 ± 8.051	−0.764^***^	−0.750^***^	0.748^***^	−0.794^***^	1

* < 0.05, ** < 0.01; *** < 0.001; PSI, Peer Support Intervention; RES, Resilience; PSR, Perceived Stress; NM, Negative Mood; PM, Positive Mood.

### 3.5 Multicollinearity diagnostics

Preliminary correlation analyses revealed significant associations among several variables, raising concerns regarding potential multicollinearity, which may bias or inflate regression estimates. To address this issue, all continuous variables in each equation were standardized, and multicollinearity diagnostics were conducted. The results showed that the tolerance values ranged from 0.256 to 0.415 (>0.1), and the variance inflation factors (VIFs) ranged from 2.412 to 3.899 (<5). These findings suggest that multicollinearity is not a concern in the current dataset, thereby supporting the validity of subsequent mediation analysis.

### 3.6 Parallel mediation model analysis

**Figure 3:**
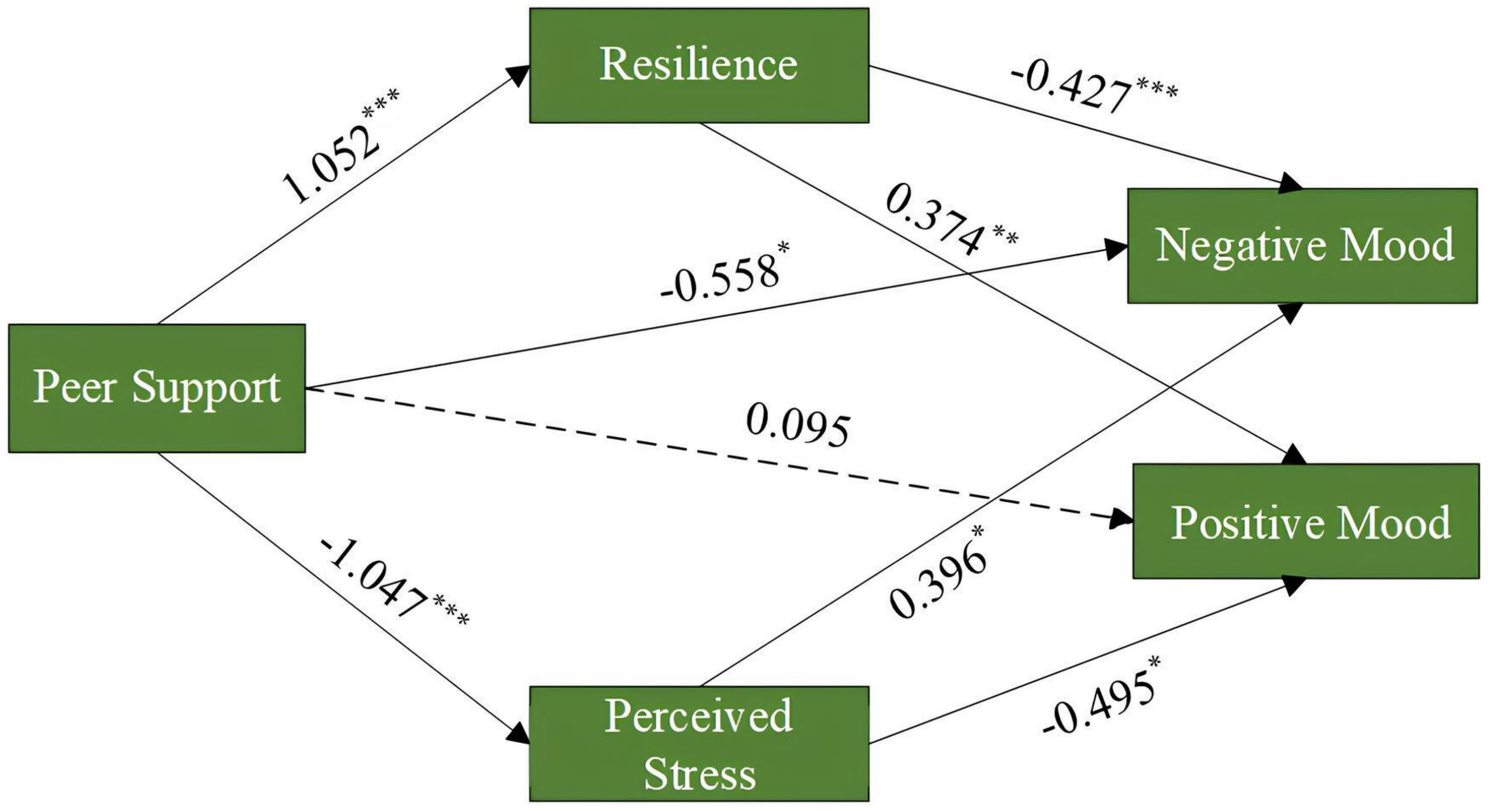
Parallel mediation model diagram.

Based on the data from post-intervention and follow-up measurements, this study used Hayes’ SPSS-Process to construct and test a parallel mediation model. Template Model 4 was selected, with the group factor as a binary independent variable, coded as “0″ for the control group and “1″ for the experimental group. Participants’ negative and positive mood states were treated as dependent variables, while psychological resilience and perceived stress served as Parallel mediators. The path coefficients are presented in Figure 3. Using the Bootstrap method with 5,000 repetitions and a default 95% confidence interval, the mediation effect was tested. The results revealed that the total effects of peer support on negative and positive mood states were −1.422 and 1.007, respectively.

Regression analysis (Table 6) shows that peer support intervention significantly improves psychological resilience (*β* = 1.052, *p* < 0.001) and significantly reduces perceived stress (*β* = −1.047, *p* < 0.001). It also has a significant negative effect on negative mood (*β* = −0.558, *p* < 0.05), but no significant direct effect on positive mood (*β* = 0.095, *p* > 0.05). Changes in psychological resilience significantly reduced negative mood (*β* = −0.427, *p* < 0.001), and enhanced positive mood (*β* = 0.374, *p* < 0.01). Higher levels of perceived stress were associated with increased negative mood (*β* = 0.396, *p* < 0.05) and reduced positive mood (*β* = −0.495, *p* < 0.05).

**Table 6:** Regression results for the parallel mediation model.

Dependent variable	Independent variable	*β*	*t*-value	*R^2^*	*F*
RES	PSI	1.052	4.966^***^	0.349	24.662
PSR		−1.047	−7.837^***^	0.572	61.421
NM	PSI	−0.558	−2.334^*^	0.724	38.531
	RES	−0.427	−3.807^***^		
	PSR	0.396	2.225^*^		
Total effect		−1.422	−7.464^***^	0.548	55.707
PM	PSI	0.095	0.382	0.600	22.030
	RES	0.374	3.196^**^		
	PSR	−0.495	−2.664^*^		
Total effect		1.007	5.150^***^	0.366	26.525

* < 0.05, ** < 0.01, *** < 0.001; PSI, Peer Support Intervention; RES, Resilience; PSR, Perceived Stress; NM, Negative Mood; PM, Positive Mood.

The Bootstrap method is currently considered the optimal technique for testing mediating effects. If the upper and lower bounds of the 95% confidence interval do not include zero, it indicates the presence of a significant mediating effect (Preacher and Hayes, 2008). The mediation analysis revealed both direct and indirect effects of peer support intervention (PSI) on two outcome variables: negative mood (NM) and positive mood (PM). The results are presented in Table 7.

**Table 7:** Bootstrap test results for the various paths of the model.

Model	*β*	Boot SE	95%CI		Proportion
			Boot LLCI	Boot ULCL	
Ind1a: PSI → RES → NM	−0.450	0.089	−0.419	−0.063	31.61%
Ind2a: PSI → PSR → NM	−0.415	0.115	−0.453	−0.001	29.15%
Direct effect	−0.558	0.239	−1.027	−0.089	39.24%
Total indirect effect	−0.864	0.113	−0.676	−0.231	60.76%
Ind1b: PSI → RES → PM	0.394	0.095	0.038	0.419	39.13%
Ind2b: PSI → PSR → PM	0.518	0.137	0.068	0.599	51.44%
Direct effect	0.095	0.250	−0.394	0.584	9.43%ns
Total indirect effect	0.912	0.125	0.310	0.808	90.57%

ns, not significant; PSI, Peer Support Intervention; RES, Resilience; PSR, Perceived Stress; NM, Negative Mood; PM, Positive Mood.

For negative mood (NM), the total effect of peer support intervention (PSI) was significant (*β* = −1.422, 95% *CI* [−1.796, −1.049]). The direct effect remained statistically significant (*β* = −0.558, 95% *CI* [−1.027, −0.089]), accounting for 39.24% of the total effect, while the remaining 60.76% was explained by indirect pathways. Among these, the PSI → RES → NM path contributed 31.61% (*β* = −0.450, 95% *CI* [−0.419, −0.063]), and the PSI → PSR → NM path explained 29.15% (*β* = −0.415, 95% *CI* [−0.453, −0.001]). Both mediation paths were statistically significant, underscoring the role of resilience (RES) and perceived stress (PSR) in mediating the effects of peer intervention on negative mood.

For positive mood (PM), the total effect of PSI was significant (*β* = 1.007, 95% *CI* [0.624, 1.391]). The indirect pathways jointly accounted for 90.57% of the total effect, indicating strong mediation effects through both resilience and perceived stress. Specifically, the PSI → PSR → PM pathway contributed the largest proportion (*β* = 0.518, 95% *CI* [0.068, 0.599], 51.44%), followed by the PSI → RES → PM pathway (*β* = 0.394, 95% *CI* [0.038, 0.419], 39.13%). Both pathways reached statistical significance, further emphasizing the critical mediating roles of perceived stress and resilience in enhancing positive emotional outcomes following peer support intervention.

Together, these results highlight the critical mediating roles of psychological resilience and perceived stress in shaping both negative and positive moods among injured collegiate athletes. The significant indirect effects observed across both models suggest that peer support interventions exert their psychological benefits partially via enhancing resilience and reducing perceived stress.

## 4 Discussion

Sports injuries are known to impact mental health through various psychological mechanisms. Compared to the general population, collegiate athletes demonstrate a stronger reliance on their athletic abilities, making injuries not only a threat to physical functionality but also a profound challenge to their athletic identity and self-efficacy (Simms et al., 2021). Although data from the control group suggest that natural recovery processes, such as the gradual alleviation of pain and functional impairment over time, can contribute to improvements in self-efficacy, which in turn partially alleviates perceived stress and negative moods, this effect remains limited and insufficient to address the comprehensive recovery needs of injured athletes (De Francisco et al., 2016). Consequently, peer support, as a targeted psychological intervention strategy, plays a crucial role in improving emotional states, alleviating perceived stress, and rebuilding psychological resilience, ultimately facilitating the comprehensive recovery of collegiate athletes following sports injuries.

### 4.1 The effects of peer support

To gain a comprehensive understanding of participants’ subjective experiences and behavioral patterns, this study supplemented the quantitative survey with a small-scale, non-standardized interview (*n* = 10), drawing on conceptual principles of interpretative phenomenological analysis (IPA) (Smith et al., 2021). Participants were encouraged to articulate their experiences and perspectives on the peer support intervention, including perceived emotional shifts and changes in cognitive and behavioral responses to stress. Narrative accounts offered valuable context for interpreting the quantitative findings by illustrating how participants subjectively experienced and interpreted the peer support intervention during their rehabilitation process.

Peer support proved highly effective in enhancing mood states. Post-intervention results revealed a significant increase in positive moods, such as feelings of activity and calmness, alongside a marked reduction in negative moods, including anxiety and depression. Analysis of variance (ANOVA) indicated that peer support interventions effectively improved the mood states of injured collegiate athletes, with effect sizes comparable to those reported in studies on mindfulness-based psychological therapies (Ajilchi et al., 2019). Peers, owing to their similar in age, social background, shared values, and life experiences, create an intervention environment characterized by greater relatability, lower psychological defenses, and stronger interactions. Participants are more likely to open up to peer supporters who are also collegiate athletes with similar experiences, allowing them to freely express their anxieties and concerns. This inherent advantage, rooted in group identification, further enhances the relevance and effectiveness of peer support in improving the mental well-being of collegiate athletes (Lloyd-Evans et al., 2014). One participant recounted experiencing self-doubt and anxiety in the early stages of the intervention due to the helplessness and isolation caused by their injury. However, encouragement from peers gradually helped them regain confidence and recognize that “these moods are normal and can be accepted and managed.” Several interviewees noted that they began to feel “greater peace and hope” during the peer support process and learned to confront the inevitable challenges of recovery with resilience. Emotional support and shared experiences among peers not only enabled participants to reevaluate their mental state and reduce psychological pressure but also instilled a sense of hope and accomplishment, reinforcing their confidence in the rehabilitation journey.

Peer support also demonstrated significant value in alleviating perceived stress, a subjective experience of external stressors that is closely associated with levels of anxiety and depression (Haraldsdottir and Watson, 2021). During interviews, many participants mentioned that listening to others’ recovery stories taught them to view stress more positively and adopt more adaptive coping strategies. One interviewee stated, “Peer counseling made me realize that I’m not the only one facing these problems, and that realization itself was a relief.” By sharing recovery experiences, offering practical advice, and fostering positive interpersonal interactions, peer support helped participants reinterpret and reassess the sources of their stress, leading to a marked reduction in perceived stress levels.

Psychological resilience serves as a critical protective mechanism for collegiate athletes in coping with sports injuries, playing a pivotal role not only in psychological rehabilitation but also in future athletic performance and life adaptation. Results from non-standardized interviews revealed that nearly all participants recognized the positive role of peer support in enhancing their resilience. One participant shared that peer encouragement helped them “start believing that I can overcome challenges step by step, even if it’s just achieving one small goal a day.” This strategy of setting and achieving incremental goals to gradually tackle recovery challenges was frequently mentioned and practiced by participants. As peer support deepened, these deliberate and long-term emotional regulation strategies were repeatedly activated in specific situations, facilitating the automation of emotion regulation processes (Öhman, 2002). Moreover, the role of peer support in rebuilding resilience appeared to extend to non-peer contexts, enabling individuals to quickly activate similar adaptive strategies when confronted with new stressors or adverse events, thereby reinforcing the development of psychological resilience (Hatcher et al., 2005). One participant reflected, “I found that when facing new stress, I can stay calm, figure out solutions, and no longer give up easily.” These findings further confirm the unique advantages and effectiveness of peer support in promoting psychological well-being, providing strong empirical evidence for its broader application in the psychological rehabilitation of collegiate athletes following sports injuries.

### 4.2 Separate mediating effects of perceived stress and resilience

This study demonstrates that peer support can significantly facilitate the psychological rehabilitation of collegiate athletes following sports injuries by alleviating perceived stress, enhancing psychological resilience, reducing negative emotions, and fostering positive emotions through emotional support, increased health information sharing, and positive role modeling. Data from the experimental group indicate that, following the peer support intervention, perceived stress scores decreased significantly (*p* < 0.01), while psychological resilience scores increased notably (*p* < 0.01), with both factors playing a critical role in psychological rehabilitation. Notably, the direct effect of peer support on positive affect was not statistically significant, which may be attributed to the inherently variable and context-sensitive nature of positive moods (Disabato et al., 2025; Jenkins et al., 2024).

Firstly, perceived stress is a key mediator in the process through which peer support influences psychological rehabilitation. The findings of this study indicate that peer support directly reduces perceived stress, which in turn significantly alleviates negative moods such as anger and depression, while also enhancing positive moods like activity and pleasure. Path analysis revealed that perceived stress significantly mediated the effects on both negative mood (*β* = 0.396, *p* < 0.05) and positive mood states (*β* = −0.495, *p* < 0.05). Previous studies suggest that peer support, through emotional companionship, experience sharing, and strategic guidance, helps participants reframe stressors, viewing them as manageable challenges rather than threats, thereby significantly lowering their perceived stress levels (King and Simmons, 2018). Particularly when the peer supporters are also athletes with similar injury experiences, this shared identity and empathetic ability not only enhance the effectiveness of the intervention, making injured athletes more receptive to advice and more likely to benefit from it, but also foster a deeper understanding, acceptance, and encouragement (Gassaway et al., 2017). Such experience-based support helps injured individuals cope more effectively with the elevated perceived stress caused by pain, uncertainty in recovery, and declines in athletic performance (Redinger et al., 2020). For example, by sharing personal recovery success stories, the counselor conveys possibilities and instills confidence in the rehabilitation process, which significantly alleviates the participant’s perceived stress. This peer relationship not only provides emotional support but also strengthens the participant’s positive perception of the recovery journey.

Moreover, psychological resilience serves as a critical mediator in the relationship between peer support and emotional improvement (*β_n_ =* −0.427*, β_p_ =* 0.374*, p < 0.01*). Enhanced resilience enables individuals to more effectively mobilize internal and external resources to address the negative moods triggered by injury and the recovery process (Goddard et al., 2021). Previous studies have shown that athletes with higher levels of resilience are more inclined to adopt problem-focused coping strategies—such as cognitive reappraisal and seeking social support—following sports injuries, thereby facilitating more rapid adaptation to physical changes and promoting a positive attitude toward recovery, ultimately improving their overall affective state (di Fronso et al., 2022; Stover et al., 2024). In this process, individuals’ appraisals of stressors tend to become more positive, reducing negative cognitive evaluations and enhancing emotional regulation and psychological adjustment (Riepenhausen et al., 2022).

Taken together, peer support exerted significant effects on both negative and positive affect through two parallel mediating pathways—perceived stress and psychological resilience—revealing a multi-path regulatory mechanism underlying its role in psychological rehabilitation. This finding underscores the complex and dynamic nature of emotional adjustment post-injury, where multiple psychological processes operate concurrently to facilitate recovery.

### 4.3 Practical implications

This study demonstrates that peer support interventions can significantly facilitate athletes’ mental well-being and promote psychological recovery by strengthening resilience and reducing perceived stress. For frontline practitioners in universities, athletic teams, and rehabilitation settings, peer support—characterized by its empathic nature, cost-efficiency, and timely responsiveness—represents an appropriate intervention for collegiate athletes in the early stages of rehabilitation, especially those who exhibit limited help-seeking motivation. To further enhance the efficacy of such interventions, it is recommended that peer support be integrated with foundational emotional regulation training and cognitive restructuring techniques, such as cognitive-behavioral therapy (Xiang et al., 2025), to strengthen athletes’ self-regulatory capacities and progressively restore positive self-concept and self-esteem (Richard et al., 2017).

Building on these findings, sport organizations and support teams may consider embedding peer support into stratified intervention models tailored to athletes’ psychological resource profiles—including resilience levels, perceived stress, and athletic identity status (Örencik et al., 2024). Within such frameworks, peer support can function as a scalable first-line response, enabling early psychological engagement while reserving more intensive clinical resources for those at higher risk. This personalized, proactive approach may help foster a more stable athletic identity, strengthen adaptive coping and stress-buffering capacities, and ultimately promote the sustainable development of psychological wellbeing.

### 4.4 Limitations and future directions

While this study revealed the significant intervention effects of peer support, it also highlighted certain limitations. First, peer support appears less effective in addressing complex, severe, or persistent psychological issues. Some participants noted experiencing negative emotional impacts after hearing about other members’ distressing experiences, which may indicate potential secondary trauma—a phenomenon commonly observed in group interventions (Yalom and Leszcz, 2020). Future intervention designs could consider incorporating desensitization protocols or promoting self-care practices to mitigate the occurrence of secondary trauma. Second, some participants reported that large group sizes limited opportunities for individual expression and interaction, particularly during the later stages of the intervention when support content was perceived as repetitive. To address this, future peer support programs could optimize group size and structure, such as limiting groups to 6–8 participants and introducing subgroup strategies to enhance the specificity and engagement of the intervention (Yalom and Leszcz, 2020). Third, although this study considered key psychological constructs such as resilience and perceived stress, it relied on a relatively linear mediation model and did not account for the complex, dynamic interplay among multiple psychosocial resources. Recent research suggests that combinations of resources—such as athletic identity, self-esteem, and perceived social support—may exert differential or compensatory effects on perceived stress (Örencik et al., 2024). Future research should adopt a person-oriented approach to examine how different psychosocial resources interact to shape individuals’ responsiveness to peer support, ultimately informing more precise and tailored intervention strategies.

## 5 Conclusion

This study investigates the application and effectiveness of peer support in the psychological rehabilitation of collegiate athletes recovering from sports injuries. It examines the impact of peer support on psychological rehabilitation through the parallel mediation paths of psychological resilience and perceived stress. The findings demonstrate that peer support, as a non-professional psychological intervention, significantly alleviates negative moods and promotes psychological recovery by enhancing psychological resilience and reducing perceived stress in collegiate athletes.

The study highlights that peer support, through emotional support, experience sharing, and strategic guidance, effectively improves psychological resilience, thereby strengthening athletes’ ability to cope with stress and adversity. Enhanced psychological resilience facilitates positive cognitive appraisals, enabling individuals to employ adaptive strategies that reduce perceived stress and alleviate negative moods stemming from sports injuries while simultaneously enhancing well-being and life satisfaction. Experimental results revealed that peer support significantly decreased scores on the Perceived Stress Scale and improved all dimensions of the Mood Survey Scale, indicating tangible benefits for participants in the experimental group. These findings are consistent with prior research, both domestic and international, suggesting that peer support interventions hold substantial potential as a component of psychological rehabilitation for collegiate athletes (Bernecker et al., 2020; Budde et al., 2018; Gillard, 2019; Mahlke et al., 2017). The results provide empirical evidence supporting the integration of peer support into rehabilitation-oriented mental health programs.

Furthermore, the study confirms that peer support, when implemented as an early intervention or complementary treatment, can complement professional psychological interventions, contributing to a comprehensive, multi-dimensional psychological support system. By offering early intervention and supportive counseling, injured athletes are more likely to accept assistance and improve their mood state during the initial stages of psychological recovery (Bryan and Arkowitz, 2015). However, the effectiveness of peer support depends on the training and compatibility of the counselors, particularly their professional knowledge, communication skills, and rapport with counselees (Watson, 2019). Thus, systematic training programs are essential to equip peer counselors with skills such as emotion recognition, active listening, and supportive communication, ensuring they possess the expertise required for effective intervention.

In conclusion, this study provides empirical evidence for the role of peer support in the psychological rehabilitation of collegiate athletes recovering from sports injuries. It also offers theoretical support for optimizing rehabilitation strategies and improving the quality of psychological health services. Future research should focus on integrating peer support with professional psychological interventions, fostering the development of multidisciplinary collaborative support systems that address athletes’ psychological needs and promote their comprehensive recovery and development.

## Data Availability

The raw data supporting the conclusions of this article will be made available by the authors, without undue reservation.
